# Crystal structure of 2-(morpholino)ethyl­ammonium picrate monohydrate

**DOI:** 10.1107/S2056989022011409

**Published:** 2023-01-01

**Authors:** Balasubramanian Chidambaranathan, Settu Sivaraj, Shanmugam Selvakumar

**Affiliations:** a PG and Research Department of Physics, Government Arts College for Men, (Autonomous), Chennai 600 035, Tamil Nadu, India; Universidad Nacional Autónoma de México, México

**Keywords:** single-crystal X-ray study, morpholine, hydrogen bond, IR, NMR, Hirshfeld surface

## Abstract

The title compound, C_6_H_15_N_2_O^+^·C_6_H_2_N_3_O_7_^−^·H_2_O, was synthesized *via* slow evaporation of an aqueous solution of picric acid with the substituted morpholine base and crystallized with one cation (C_6_H_15_N_2_O)^+^, one anion (C_6_H_2_N_3_O_7_)^−^ and a water mol­ecule in the asymmetric unit. The morpholine ring in the cation adopts a chair conformation. The structure is stabilized by C—H⋯O, O—H⋯O, O—H⋯N and N—H⋯O hydrogen-bonding inter­actions and π–π stacking. The inter­molecular inter­actions of the synthesized compound were qu­anti­fied by Hirshfeld surface analysis.

## Abstract 2

The title compound was synthesized *via* slow evaporation of an aqueous solution of picric acid with the substituted morpholine base and crystallized with one cation (C_6_H_15_N_2_O)^+^, one anion (C_6_H_2_N_3_O_7_)^−^ and a water mol­ecule in the asymmetric unit. The morpholine ring in the cation adopts a chair conformation. The structure is stabilized by C–H⋯O, O—H⋯O, O—H⋯N and N—H⋯O hydrogen-bonding inter­actions and π–π stacking. The inter­molecular inter­actions of the synthesized compound were qu­anti­fied by Hirshfeld surface analysis.

## 1. Chemical context

Morpholine complex materials are widely used in biomedical applications as this moiety serves as an important lysosome-targeting group. Its applications include use in the synthesis of lysosome-targetable fluorescent probes for hydrogen sulfide imaging in living cells. Morpholine can be used as a ligand in metal complexes. It is also a component of protective coatings on fresh fruits and is used as an emulsifier in the preparation of pharmaceuticals and cosmetic products (Kuchowicz & Rydzyński, 1998 ▸). Picric acid forms stable picrates with various organic mol­ecules through bonding or ionic bonding. It is also a well-established material for non-linear optical (NLO) substances, which crystallize in the non-centrosymmetric space group *Pca*2_1_ (Yamaguchi *et al.*, 1988 ▸). Compounds of the morpholine family such as 4-(2-chloro­eth­yl)morpholinium picrate (Kant *et al.*, 2009 ▸), 4-(4-nitro­phen­yl)morpholine (Wang *et al.*, 2012 ▸), morpholinium picrate (Vembu & Fronczek, 2009 ▸) can be used in drug design. The phenolic group of the picrate anion might favour the formation of hydrogen-bonding inter­actions to increase the mol­ecular hyperpolarizability and NLO effects (Takayanagi *et al.*, 1996 ▸). Organic mol­ecules have attracted great attention because of their ability to combine low cost and ease of processing in the assembly of optical devices. In this context, the present investigation reports the synthesis, crystal structure, Hirshfeld surface, IR and NMR analyses of 2-(morph­olin­yl)ethyl­ammonium picrate monohydrate. 
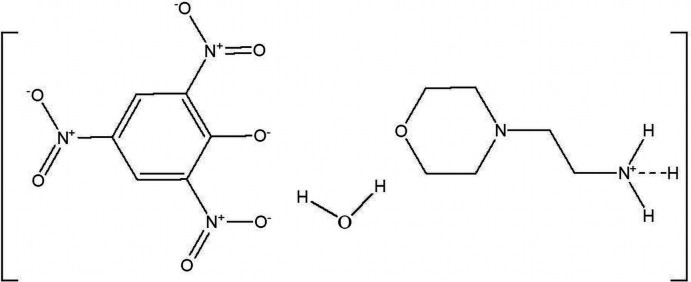


## 2. Structural commentary

**Figure 1:**
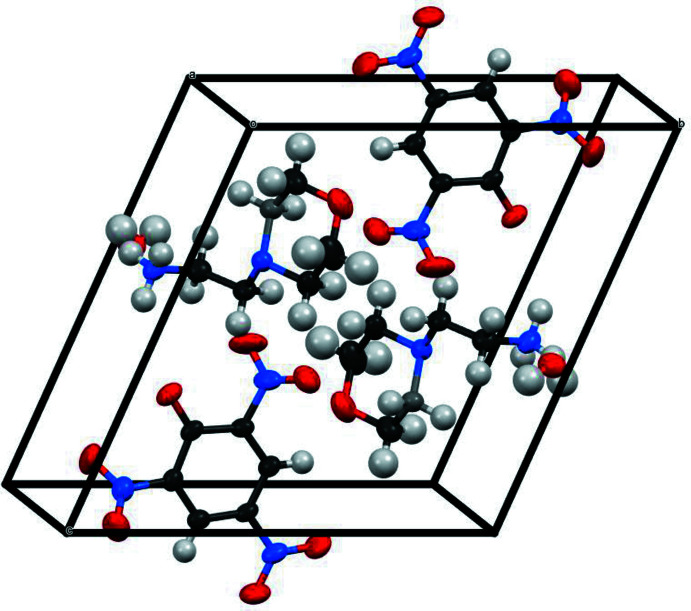
Mol­ecular diagram of the title compound viewed down along a* axis in the unit cell.

**Figure 2:**
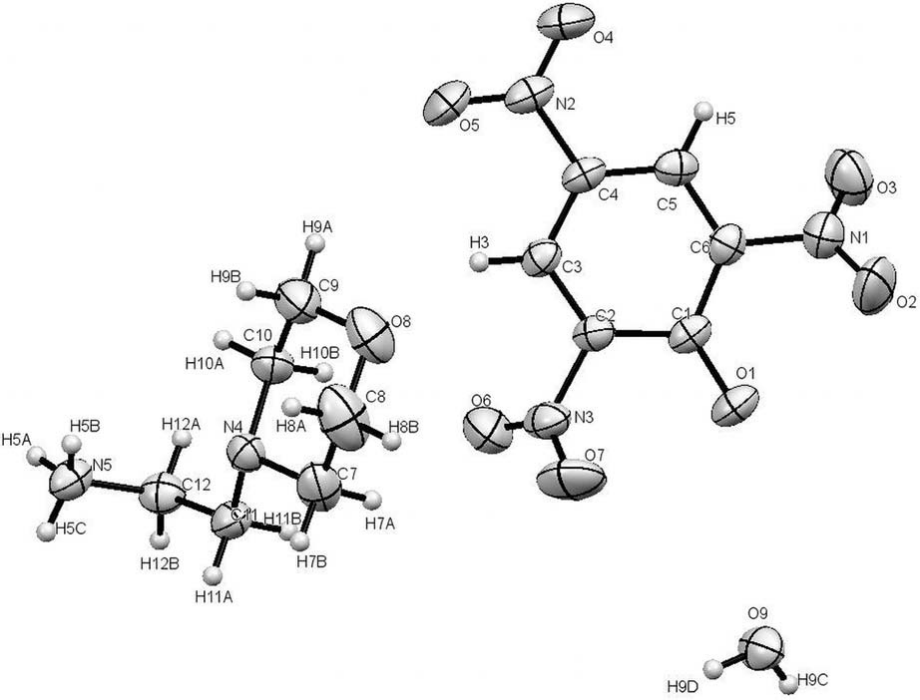
*ORTEP* diagram of the title compound with the atom-numbering scheme. Displacement ellipsoids are drawn at the 50% probability level.

The title compound crystallizes in the triclinic *P* 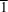 space group (Fig. 1 ▸) with two ion pairs and two solvating water mol­ecules in the unit cell (Fig. 1 ▸). The asymmetric unit is shown in Fig. 2 ▸. In agreement with the p*K*_a_ constants for the parent 4-(2-ammonio­eth­yl)morpholine (4.84, 9.45), the terminal NH_2_ group of the base is protonated, forming the 2-(morpholino)­ethyl­ammonium anion. All three protons of the NH_3_^+^ group are involved in hydrogen bonding. The cation forms a strong charge-assisted hydrogen bond N5—H5*A*⋯O1 [1 − *x*, −*y*, 1 − *z*; *D*⋯*A* = 2.777 (2) Å] with the picrate anion, while H5*C* inter­acts with O9 from the solvating water mol­ecule [*D*⋯*A =* 2.741 (2) Å] and H5*B* is involved in a bifurcated hydrogen bond with O5 from a neighbouring picrate anion [−1 + *x*, −1 + *y*, −1 + *z*; *D*⋯*A* = 3.054 (2) Å] and O9 from other water mol­ecule (−*x*, −*y*, −*z* + 1), respectively. Additionally, the two protons of the water mol­ecule inter­act with a picrate anion or the nitro­gen atom of the morpholinyl moiety [O9—H9*C*⋯O1, *D*⋯*A* = 2.7196 (19) Å; O9—H9*D*⋯N4, −*x*, −*y*, −*z* + 1, *D*⋯*A* = 2.7722 (19) Å]. Further geometric details of these hydrogen bonds can be found in Table 1 ▸. In this scenario, the water mol­ecule forms a bridge between the ammonium group and another picrate anion that cannot inter­act directly for steric reasons. Formation of these hydrogen bonds also lowers the energy of the crystal and thus increases the stability of the packing.

The refined geometry (Fig. 2 ▸) shows that the torsion angles N4—C7—C8—O8 and O8—C9—C10—N4 of the morpholine ring are −58.1 (2) and 59.4 (2)°, respectively, confirming the chair conformation. There are three nitro groups in the picrate anion. While the *para-*bound nitro group is nearly coplanar with the plane of the benzene ring [dihedral angle of −1.0 (2)°] and two *ortho-*oriented nitro groups are, probably as a result of repulsion with the phenolic oxygen atom, twisted from the ring plane by −51.9 (2) and 43.8 (2)°. It has been mentioned previously that the nitro groups of the picrate anion play an important role in stabilizing the crystal packing *via* weak coulombic inter­actions (George *et al.*, 2019 ▸; Anitha *et al.*, 2004 ▸).

## 3. Supra­molecular features

**Figure 3:**
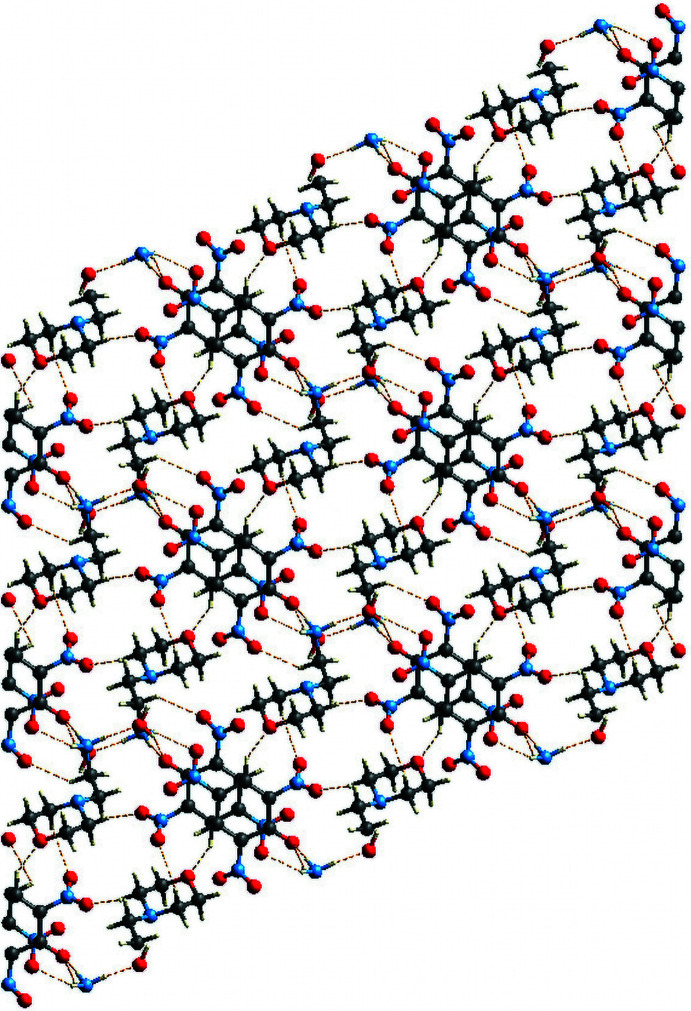
Three-dimensional supra­molecular architecture of the title compound viewed down the *a* axis.

**Figure 4:**
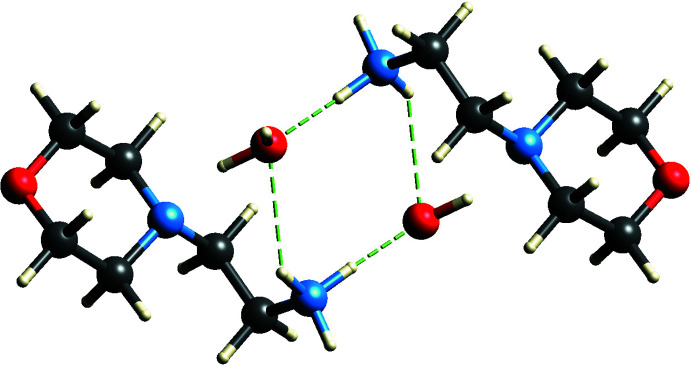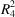 (8) ring motif formed between ammonium group and water mol­ecules.

**Figure 5:**
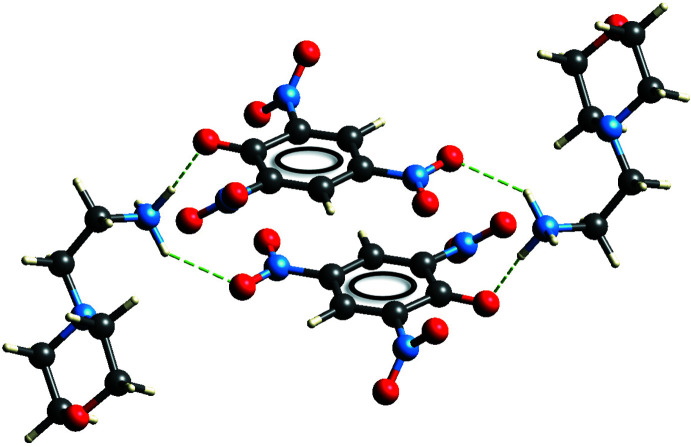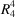 (20) ring motif formed between two ammonium group and two picrate anions and π–π stacking inter­actions.

**Figure 6:**
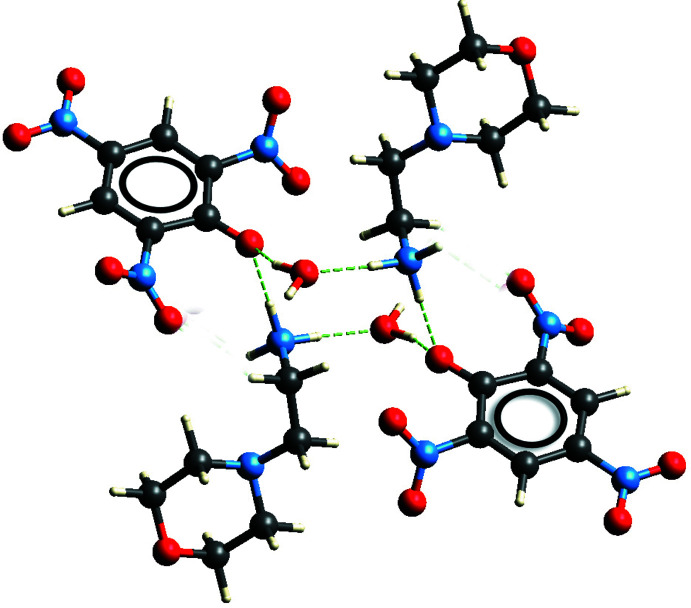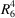 (12) ring motif formed through twelve-membered ring [(picrate)O^−^⋯H—N—H⋯O—H]_2_ inter­actions.

Fig. 3 ▸ shows the three-dimensional mol­ecular packing of the title compound viewed down the *a*-axis. Along with the six main hydrogen bonds described in the previous section, the cation inter­acts with neighboring picrate anions *via* C7—H7*B*⋯O7(*x* − 1, *y*, *z*), C10—H10*B*⋯O6(−*x* + 1, −*y* + 1, −*z* + 1) and C12—H12*A*⋯O2(−*x* + 1, −*y*, −*z* + 1) non-classical hydrogen bonds (Table 1 ▸). Several prominent supra­molecular motifs are formed by these hydrogen bonds. Firstly, the inter­action of the ammonium group with the water mol­ecules creates a centrosymmetric motif described by an 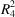 (8) graph set (Bernstein *et al.*, 1995 ▸; Motherwell *et al.*, 2000 ▸) (Fig. 4 ▸). Next, another centrosymmetric motif described by an 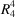 (20) graph set is formed between two ammonium groups and two picrate anions and involves the phenolic oxygen anion and the *para* nitro group (Fig. 5 ▸). Furthermore, the picrate anions are coplanar, and are involved in two different π–π stacking inter­actions with perpendicular distances between the C1–C6 rings of 3.3532 (6) and 3.5533 (6) Å, slippage of 1.393 and 1.902 Å, and *Cg*⋯*Cg* distances of 3.6311 (18) and 4.0303 (19) Å, respectively, for the rings related by symmetry operations 1 − *x*, 1 − *y*, 2 − *z* and 2 − *x*, 1 − *y*, 2 − *z*. Finally, a centrosymmetric twelve-membered ring [(picrate)O^−^⋯H—N—H⋯O—H]_2_ with a third order graph set 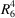 (12) involves two of each of the three different species present in the crystal (Fig. 6 ▸).

**Figure 7:**
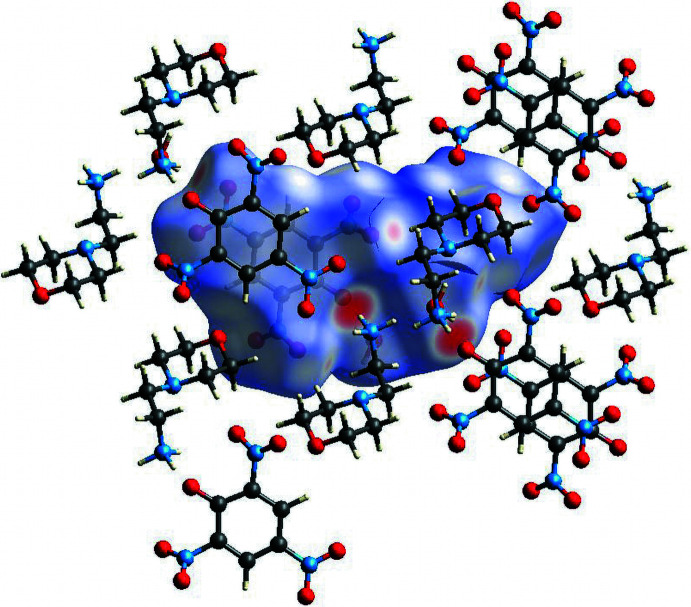
The Hirshfeld surface of the title compound mapped over *d*_norm_, showing the closest mol­ecules.

Analysis of the Hirshfeld surface and the associated two-dimensional fingerprint plot for 2-(morpholin­yl)ethyl­ammonium picrate monohydrate was performed with *CrystalExplorer 21.5* (Spackman *et al.*, 2021 ▸). The normalized contact distance (*d*_norm_) Hirshfeld surface of the title compound mapped over the limits −0.6471 to 1.3714 a.u. with close contacts to neighboring mol­ecules is shown in Fig. 7 ▸. The contacts with distances equal to the sum of the van der Waals radii are indicated in white and the contacts with distances shorter than and longer than van der Waals radii are represented as red and blue, respectively (Venkatesan *et al.*, 2016 ▸). This analysis confirms that the most prominent inter­molecular inter­actions present in the crystal are C—H⋯O, N—H⋯O, O—H⋯O and N—O⋯H contacts.

**Figure 8:**
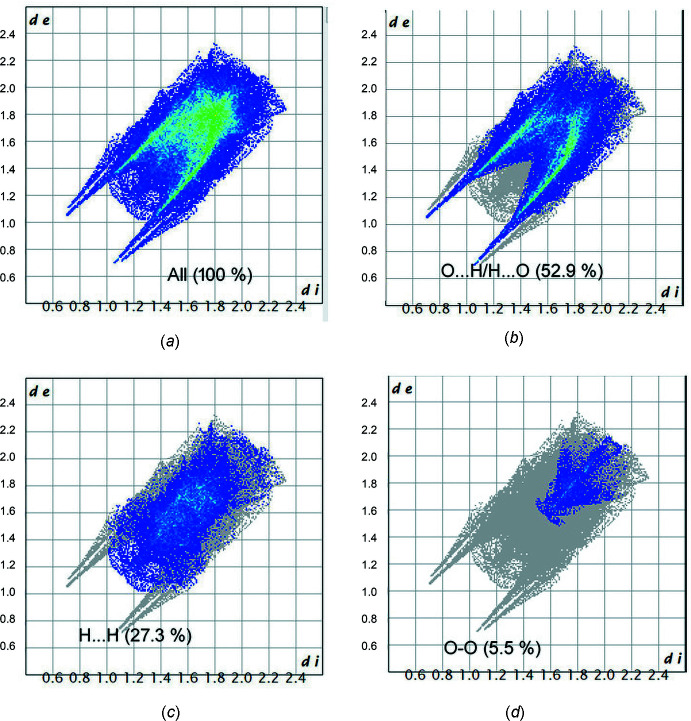
Two-dimensional fingerprint plots for the title compound showing (*a*) all inter­actions, (*b*) O⋯H/H⋯O inter­actions, (*c*) H⋯·H inter­actions and (*d*) O⋯O inter­actions.

Two-dimensional fingerprint plots of the sum of the contacts contributing to the Hirshfeld surface represented in normal mode are shown in Fig. 8 ▸. In the figure, *d*_e_ and *d*_i_ represent the distances from a point on the Hirshfeld surface to the nearest atoms outside and inside the surface, respectively (McKinnon *et al.*, 2007 ▸; Seth, 2014 ▸; Nchioua *et al.*, 2022 ▸). The most significant contribution to the Hirshfeld surface is from the O⋯H/H⋯O (52.9%) inter­actions. In addition, the H⋯H (27.3%) and O⋯O (5.5%) inter­actions make significant contributions to the total Hirshfeld surface. Other inter­actions contributing less than 5.0% are C⋯C (3.9%), O⋯C/C⋯O (2.5%), N⋯H/H⋯N (2.2%), N⋯C/C⋯N (2.2%), H⋯C/C⋯H (1.8%) and O⋯N/N⋯O (1.8%).

## 4. Database survey

A search in the Cambridge Structural Database (CSD, version 5.40; Groom *et al.*, 2016 ▸) found eleven structures containing 4-(2-ammonio­eth­yl)morpholinium including 4-(2-ammonio­eth­yl)morpholinium tetra­chloro­copper(II) (BOPWUY and BOPWUY01; Battaglia *et al.*, 1982 ▸), 4-(2-ammonium­eth­yl)morpholinium tetra­chloro­mercury(II) (CUMGIA; Vezzosi *et al.*, 1984 ▸), 4-(2-ammonio­eth­yl)morpholinium dichloride mono­hydrate (JAXBOC; Ghorab *et al.*, 2017 ▸), 4-(2-ammo­nio­eth­yl)morpholinium tetra­chloro­palladium(II) (KETHOJ; Efimenko *et al.*, 2017 ▸), 4-(2-ammonio­eth­yl)morpholinium sulfate methanol solvate (KUTZUV; Bi, 2010 ▸), *catena*-[4-(2-ammonio­eth­yl)morpholinium] tetra­kis­[(μ_3_-phosphito)tri­zinc(II)] hemihydrate (SEZPOE; Lin & Dehnen, 2009 ▸), 4-(2-ammonio­eth­yl)morpholinium di­chloro­diiodo­cadmium(II) chloro­tri­iodo­cadmium(II) (UVWEZ; Mahbouli Rhouma *et al.*, 2016 ▸), 4-(2-ammonio­eth­yl)morpholinium tetra­chloro­zinc(II) (WUTGOI; Glaoui *et al.*, 2008 ▸ and WUTGOI01; Lamshöft *et al.*, 2011 ▸), *catena*-[bis­[4-(2-ammonio­eth­yl)morph­o­linium] tetra­kis­(μ-iodo)­tetra­kis­(iodo)­dilead(II)] and (NIXNEQ; Xiuli & Zhenhong, 2019 ▸). Unlike the title compound, all of these examples have both nitro­gen atoms protonated. Another search in the CSD for the compound morpholinium picrate gave four hits, *viz*. 4-hy­droxy-4-methyl­morpholinium picrate (HIGYOM; Zukerman-Schpector *et al.*, 2007 ▸), morpholinium picrate (KOMTUC; Vembu & Fronczek, 2009 ▸), 4-(2-chloro­eth­yl)morpholinium picrate (PUFFIG; Kant *et al.*, 2009 ▸) and 4,4-bis­(2′-hy­droxy­eth­yl)morpholinium picrate (SEGGAM; Solov’ev *et al.*, 1988 ▸). It is noted that all of these structures are stabilized by hydrogen bonds and that in each one the morpholine ring has a chair conformation.

## 5. Synthesis and crystallization

**Figure 9:**
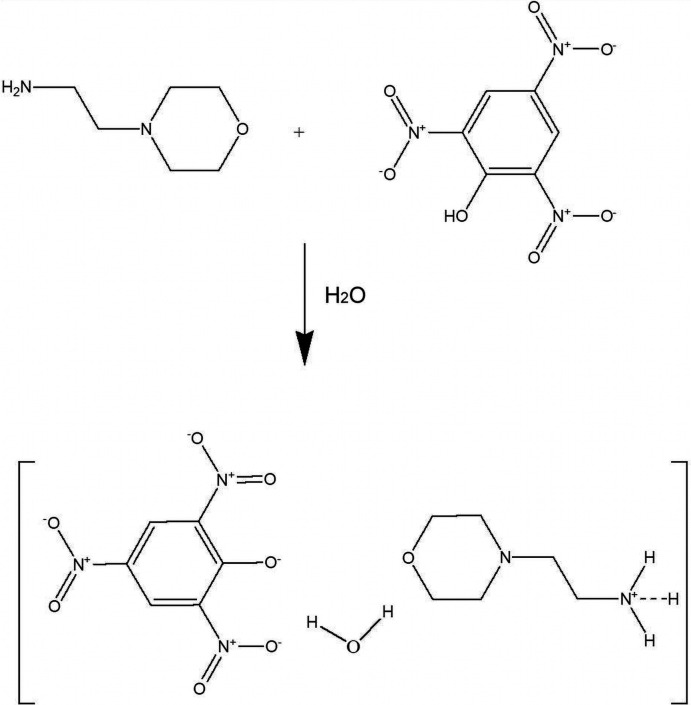
Reaction scheme for the title compound.

2-(Morpholin­yl)ethyl­ammonium picrate monohydrate was synthesized by mixing one mole of 4-(2-ammonio­eth­yl)morpholine and one mole of picric acid in double-distilled water at about 303 K. The solution was then allowed to evaporate at room temperature, which yielded yellow plate-like crystals of 2-(morpholin­yl)ethyl­ammonium picrate monohydrate. The reaction scheme is shown in Fig. 9 ▸. Melting point: 457–459 K; IR (KBr, cm^−1^): 3384 (O—H), 2905 (NH_3_), 3110 (C—H), 1382 (CH_2_), 993 (C—O); ^1^H NMR (500 MHz, D_2_O, δ, ppm): 8.831 (*s*, 2H, picrate moiety), 3.63 (*t*, 4H, –C*H*_2_–O–C*H*_2_), 3.03 (*t*, 4H, –C*H*_2_–N–C*H*_2_), 2.58 (*t*, 2H, N–C*H*_2_), 2.46 (*t*, 2H, –CH_2_–NH_3_^+^). A suitable single crystal of 2-(morpholin­yl)ethyl­ammonium picrate monohydrate was selected for X-ray diffraction studies.

## 6. Refinement

Crystal data, data collection and structure refinement details are summarized in Table 2 ▸. The C-bound H atoms were positioned geometrically (C—H = 0.93 for anion and 0.97 Å for cation) and refined using an isotropic approximation, with *U*_iso_(H) = 1.2 *U*_eq_(C). The acidic protons were localized from the residual electron-density map and refined with distance restraints (0.82 Å for O—H and 0.86 Å for N—H) and *U*_iso_(H) = 1.2*U*_eq_(N) and 1.5*U*_eq_(O).

## Supplementary Material

Crystal structure: contains datablock(s) I. DOI: 10.1107/S2056989022011409/jq2022sup1.cif

Structure factors: contains datablock(s) I. DOI: 10.1107/S2056989022011409/jq2022Isup3.hkl

Supporting information file. DOI: 10.1107/S2056989022011409/jq2022Isup3.cml

CCDC reference: 2222322

Additional supporting information:  crystallographic information; 3D view; checkCIF report

## Figures and Tables

**Table 1:** Hydrogen-bond geometry (Å, °)

*D*—H⋯*A*	*D*—H	H⋯*A*	*D*⋯*A*	*D*—H⋯*A*
C7—H7*B*⋯O7^i^	0.97	2.55	3.465 (2)	158
C10—H10*B*⋯O6^ii^	0.97	2.63	3.503 (3)	150
C12—H12*A*⋯O2^iii^	0.97	2.55	3.349 (3)	139
N5—H5*C*⋯O9	0.89 (2)	1.86 (2)	2.741 (2)	172 (2)
N5—H5*A*⋯O1^iii^	0.89 (2)	1.89 (2)	2.777 (2)	172 (2)
N5—H5*B*⋯O5^iv^	0.85 (2)	2.35 (2)	3.054 (2)	142 (2)
N5—H5*B*⋯O9^v^	0.85 (2)	2.48 (2)	3.048 (2)	126 (2)
O9—H9*D*⋯N4^v^	0.84 (2)	1.98 (2)	2.7722 (19)	158 (2)
O9—H9*C*⋯O1	0.82 (2)	1.94 (2)	2.7196 (19)	160 (2)

Symmetry codes: (i) 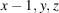 ; (ii) 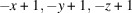 ; (iii) 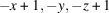 ; (iv) 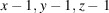 ; (v) 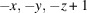 .

**Table 2:** Experimental details

Crystal data	
Chemical formula	C_6_H_15_N_2_O^+^·C_6_H_2_N_3_O_7_·H_2_O
*M*_r_	377.32
Crystal system, space group	Triclinic, *P* 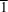
Temperature (K)	297
*a*, *b*, *c* (Å)	6.938 (3), 11.583 (5), 12.077 (5)
α, β, γ (°)	114.362 (13), 94.261 (14), 103.841 (15)
*V* (Å^3^)	841.8 (6)
*Z*	2
Radiation type	Mo *K*α
μ (mm^−1^)	0.13
Crystal size (mm)	0.40 × 0.38 × 0.19

Data collection	
Diffractometer	Bruker D8 Venture Diffractometer
Absorption correction	Multi-scan (*SADABS*; Bruker 2016 ▸)
*T*_min_, *T*_max_	0.504, 0.562
No. of measured, independent and observed [*I* > 2σ(*I*)] reflections	19297, 3378, 2781
*R*_int_	0.039
(sin θ/λ)_max_ (Å^−1^)	0.626

Refinement	
*R*[*F* ^2^ > 2σ(*F* ^2^)], *wR*(*F* ^2^), *S*	0.041, 0.117, 1.04
No. of reflections	3378
No. of parameters	250
No. of restraints	5
H-atom treatment	H atoms treated by a mixture of independent and constrained refinement
Δρ_max_, Δρ_min_ (e Å^−3^)	0.25, −0.21

Computer programs: *APEX3*, *SAINT* and *XPREP* (Bruker, 2016 ▸), *SHELXT2018/2* (Sheldrick, 2015*a* ▸), *SHELXL2018/3* (Sheldrick, 2015*b* ▸), *ORTEP-3 for Windows* (Farrugia, 2012 ▸) and *Mercury* (Macrae *et al.*, 2020 ▸).

## supplementary crystallographic information

### Crystal data

**Table:**

C_6_H_15_N_2_O^+^·C_6_H_2_N_3_O_7_·H_2_O	*Z* = 2
*M**_r_* = 377.32	*F*(000) = 396
Triclinic, *P*1	*D*_x_ = 1.489 Mg m^−^^3^
*a* = 6.938 (3) Å	Mo *K*α radiation, λ = 0.71073 Å
*b* = 11.583 (5) Å	Cell parameters from 7824 reflections
*c* = 12.077 (5) Å	θ = 3.2–26.2°
α = 114.362 (13)°	µ = 0.13 mm^−^^1^
β = 94.261 (14)°	*T* = 297 K
γ = 103.841 (15)°	Block, yellow
*V* = 841.8 (6) Å^3^	0.40 × 0.38 × 0.19 mm

### Data collection

**Table:**

Bruker D8 Venture Diffractometer	2781 reflections with *I* > 2σ(*I*)
Radiation source: fine focus sealed tube	*R*_int_ = 0.039
φ and ω scans	θ_max_ = 26.4°, θ_min_ = 3.1°
Absorption correction: multi-scan (SADABS; Bruker 2016)	*h* = −8→8
*T*_min_ = 0.504, *T*_max_ = 0.562	*k* = −14→14
19297 measured reflections	*l* = −15→15
3378 independent reflections	

### Refinement

**Table:**

Refinement on *F*^2^	5 restraints
Least-squares matrix: full	Hydrogen site location: mixed
*R*[*F*^2^ > 2σ(*F*^2^)] = 0.041	H atoms treated by a mixture of independent and constrained refinement
*wR*(*F*^2^) = 0.117	*w* = 1/[σ^2^(*F*_o_^2^) + (0.0542*P*)^2^ + 0.2721*P*] where *P* = (*F*_o_^2^ + 2*F*_c_^2^)/3
*S* = 1.04	(Δ/σ)_max_ < 0.001
3378 reflections	Δρ_max_ = 0.25 e Å^−^^3^
250 parameters	Δρ_min_ = −0.21 e Å^−^^3^

### Special details

**Table:**

Geometry. All esds (except the esd in the dihedral angle between two l.s. planes) are estimated using the full covariance matrix. The cell esds are taken into account individually in the estimation of esds in distances, angles and torsion angles; correlations between esds in cell parameters are only used when they are defined by crystal symmetry. An approximate (isotropic) treatment of cell esds is used for estimating esds involving l.s. planes.

### Fractional atomic coordinates and isotropic or equivalent isotropic displacement parameters (Å^2^)

**Table:**

	*x*	*y*	*z*	*U*_iso_*/*U*_eq_	
C1	0.6187 (2)	0.30558 (14)	0.82290 (13)	0.0335 (3)	
C2	0.6867 (2)	0.43310 (15)	0.82356 (13)	0.0349 (3)	
C3	0.7623 (2)	0.55224 (14)	0.92800 (13)	0.0355 (3)	
H3	0.802400	0.631735	0.922088	0.043*	
C4	0.7771 (2)	0.55051 (14)	1.04247 (13)	0.0336 (3)	
C5	0.7107 (2)	0.43331 (15)	1.05278 (13)	0.0360 (3)	
H5	0.716562	0.434004	1.130246	0.043*	
C6	0.6359 (2)	0.31567 (14)	0.94612 (14)	0.0359 (3)	
C7	0.0106 (3)	0.30407 (19)	0.38759 (18)	0.0542 (5)	
H7A	0.121632	0.382644	0.406388	0.065*	
H7B	−0.037972	0.314606	0.463429	0.065*	
C8	−0.1579 (4)	0.2906 (3)	0.2924 (2)	0.0739 (6)	
H8A	−0.272185	0.215447	0.277962	0.089*	
H8B	−0.201836	0.369717	0.324210	0.089*	
C9	−0.0362 (3)	0.1539 (2)	0.13116 (18)	0.0581 (5)	
H9A	0.003134	0.139093	0.052136	0.070*	
H9B	−0.150414	0.078921	0.117385	0.070*	
C10	0.1378 (3)	0.16274 (16)	0.21970 (14)	0.0432 (4)	
H10A	0.174467	0.080947	0.185378	0.052*	
H10B	0.254271	0.234838	0.230153	0.052*	
C11	0.2592 (3)	0.20986 (16)	0.43386 (14)	0.0425 (4)	
H11A	0.214563	0.221718	0.511030	0.051*	
H11B	0.358912	0.292112	0.449159	0.051*	
C12	0.3601 (2)	0.10098 (17)	0.39727 (15)	0.0438 (4)	
H12A	0.408246	0.090738	0.321368	0.053*	
H12B	0.477074	0.128043	0.461345	0.053*	
N1	0.5579 (2)	0.19374 (14)	0.95933 (14)	0.0490 (4)	
N2	0.8605 (2)	0.67574 (14)	1.15356 (12)	0.0428 (3)	
N3	0.6630 (2)	0.43738 (14)	0.70341 (12)	0.0476 (4)	
N4	0.08400 (19)	0.18564 (12)	0.34122 (11)	0.0370 (3)	
N5	0.2271 (2)	−0.02941 (14)	0.37799 (13)	0.0420 (3)	
H5C	0.206 (3)	−0.0270 (19)	0.4503 (15)	0.050*	
H5A	0.291 (3)	−0.0899 (17)	0.3458 (17)	0.050*	
H5B	0.117 (2)	−0.0515 (19)	0.3291 (16)	0.050*	
O1	0.54301 (17)	0.19894 (10)	0.72393 (10)	0.0447 (3)	
O2	0.5959 (3)	0.09346 (14)	0.88953 (15)	0.0832 (5)	
O3	0.4599 (2)	0.19858 (15)	1.03953 (15)	0.0684 (4)	
O4	0.8730 (3)	0.67488 (14)	1.25430 (11)	0.0688 (4)	
O5	0.9152 (2)	0.77882 (12)	1.14134 (12)	0.0644 (4)	
O6	0.5699 (3)	0.50981 (15)	0.69044 (13)	0.0705 (4)	
O7	0.7341 (3)	0.36689 (18)	0.62204 (13)	0.0786 (5)	
O8	−0.0951 (2)	0.27241 (15)	0.17867 (14)	0.0665 (4)	
O9	0.19445 (19)	−0.00465 (13)	0.61101 (11)	0.0519 (3)	
H9D	0.137 (3)	−0.055 (2)	0.640 (2)	0.078*	
H9C	0.293 (3)	0.055 (2)	0.661 (2)	0.078*	

### Atomic displacement parameters (Å^2^)

**Table:**

	*U*^11^	*U*^22^	*U*^33^	*U*^12^	*U*^13^	*U*^23^
C1	0.0244 (7)	0.0322 (7)	0.0346 (7)	0.0087 (5)	0.0061 (5)	0.0060 (6)
C2	0.0309 (7)	0.0369 (8)	0.0294 (7)	0.0056 (6)	0.0067 (5)	0.0100 (6)
C3	0.0317 (7)	0.0308 (7)	0.0358 (7)	0.0044 (6)	0.0063 (6)	0.0098 (6)
C4	0.0277 (7)	0.0330 (7)	0.0290 (7)	0.0096 (6)	0.0031 (5)	0.0036 (6)
C5	0.0344 (7)	0.0420 (8)	0.0321 (7)	0.0170 (6)	0.0075 (6)	0.0136 (6)
C6	0.0332 (7)	0.0331 (7)	0.0411 (8)	0.0128 (6)	0.0086 (6)	0.0144 (6)
C7	0.0680 (12)	0.0527 (10)	0.0598 (11)	0.0313 (9)	0.0337 (9)	0.0311 (9)
C8	0.0679 (13)	0.0920 (17)	0.1018 (18)	0.0464 (13)	0.0368 (13)	0.0651 (15)
C9	0.0679 (12)	0.0549 (11)	0.0506 (10)	0.0109 (9)	0.0020 (9)	0.0282 (9)
C10	0.0536 (10)	0.0408 (8)	0.0351 (8)	0.0137 (7)	0.0126 (7)	0.0163 (7)
C11	0.0480 (9)	0.0355 (8)	0.0338 (7)	0.0058 (7)	0.0037 (6)	0.0102 (6)
C12	0.0360 (8)	0.0482 (9)	0.0415 (8)	0.0089 (7)	0.0031 (6)	0.0174 (7)
N1	0.0560 (9)	0.0392 (8)	0.0507 (8)	0.0138 (6)	0.0090 (7)	0.0195 (6)
N2	0.0392 (7)	0.0411 (8)	0.0327 (7)	0.0122 (6)	0.0005 (5)	0.0028 (6)
N3	0.0478 (8)	0.0444 (8)	0.0316 (7)	−0.0044 (6)	0.0051 (6)	0.0092 (6)
N4	0.0404 (7)	0.0353 (7)	0.0366 (6)	0.0106 (5)	0.0110 (5)	0.0170 (5)
N5	0.0398 (7)	0.0380 (7)	0.0397 (7)	0.0140 (6)	0.0005 (6)	0.0091 (6)
O1	0.0401 (6)	0.0337 (6)	0.0401 (6)	0.0054 (5)	0.0035 (5)	0.0012 (5)
O2	0.1348 (15)	0.0412 (8)	0.0806 (11)	0.0375 (9)	0.0376 (10)	0.0248 (7)
O3	0.0757 (10)	0.0671 (9)	0.0775 (10)	0.0192 (7)	0.0320 (8)	0.0447 (8)
O4	0.0949 (11)	0.0606 (9)	0.0300 (6)	0.0171 (8)	−0.0013 (6)	0.0059 (6)
O5	0.0817 (10)	0.0347 (7)	0.0472 (7)	−0.0018 (6)	−0.0032 (6)	0.0034 (5)
O6	0.0939 (11)	0.0636 (9)	0.0541 (8)	0.0165 (8)	−0.0004 (7)	0.0324 (7)
O7	0.0844 (11)	0.0975 (12)	0.0404 (7)	0.0240 (9)	0.0276 (7)	0.0171 (7)
O8	0.0720 (9)	0.0768 (10)	0.0768 (9)	0.0316 (8)	0.0178 (7)	0.0532 (8)
O9	0.0455 (7)	0.0502 (7)	0.0456 (7)	−0.0059 (5)	0.0019 (5)	0.0199 (6)

### Geometric parameters (Å, º)

**Table:**

C1—O1	1.2683 (18)	C9—H9B	0.9700
C1—C2	1.435 (2)	C10—N4	1.4736 (19)
C1—C6	1.437 (2)	C10—H10A	0.9700
C2—C3	1.375 (2)	C10—H10B	0.9700
C2—N3	1.471 (2)	C11—N4	1.477 (2)
C3—C4	1.387 (2)	C11—C12	1.511 (2)
C3—H3	0.9300	C11—H11A	0.9700
C4—C5	1.385 (2)	C11—H11B	0.9700
C4—N2	1.4547 (19)	C12—N5	1.482 (2)
C5—C6	1.378 (2)	C12—H12A	0.9700
C5—H5	0.9300	C12—H12B	0.9700
C6—N1	1.465 (2)	N1—O3	1.214 (2)
C7—N4	1.482 (2)	N1—O2	1.225 (2)
C7—C8	1.509 (3)	N2—O4	1.2175 (19)
C7—H7A	0.9700	N2—O5	1.236 (2)
C7—H7B	0.9700	N3—O7	1.216 (2)
C8—O8	1.420 (3)	N3—O6	1.224 (2)
C8—H8A	0.9700	N5—H5C	0.887 (15)
C8—H8B	0.9700	N5—H5A	0.889 (15)
C9—O8	1.426 (3)	N5—H5B	0.845 (15)
C9—C10	1.507 (3)	O9—H9D	0.836 (16)
C9—H9A	0.9700	O9—H9C	0.821 (17)
			
O1—C1—C2	122.65 (14)	N4—C10—H10A	109.4
O1—C1—C6	125.22 (14)	C9—C10—H10A	109.4
C2—C1—C6	112.03 (12)	N4—C10—H10B	109.4
C3—C2—C1	125.19 (14)	C9—C10—H10B	109.4
C3—C2—N3	117.32 (14)	H10A—C10—H10B	108.0
C1—C2—N3	117.40 (13)	N4—C11—C12	114.81 (13)
C2—C3—C4	118.10 (14)	N4—C11—H11A	108.6
C2—C3—H3	120.9	C12—C11—H11A	108.6
C4—C3—H3	120.9	N4—C11—H11B	108.6
C5—C4—C3	121.52 (13)	C12—C11—H11B	108.6
C5—C4—N2	119.86 (14)	H11A—C11—H11B	107.5
C3—C4—N2	118.61 (14)	N5—C12—C11	114.31 (14)
C6—C5—C4	118.77 (14)	N5—C12—H12A	108.7
C6—C5—H5	120.6	C11—C12—H12A	108.7
C4—C5—H5	120.6	N5—C12—H12B	108.7
C5—C6—C1	124.34 (14)	C11—C12—H12B	108.7
C5—C6—N1	117.80 (14)	H12A—C12—H12B	107.6
C1—C6—N1	117.76 (13)	O3—N1—O2	123.96 (16)
N4—C7—C8	110.78 (17)	O3—N1—C6	117.76 (14)
N4—C7—H7A	109.5	O2—N1—C6	118.28 (15)
C8—C7—H7A	109.5	O4—N2—O5	122.81 (14)
N4—C7—H7B	109.5	O4—N2—C4	118.89 (15)
C8—C7—H7B	109.5	O5—N2—C4	118.30 (14)
H7A—C7—H7B	108.1	O7—N3—O6	123.84 (16)
O8—C8—C7	111.62 (16)	O7—N3—C2	117.99 (17)
O8—C8—H8A	109.3	O6—N3—C2	118.16 (14)
C7—C8—H8A	109.3	C10—N4—C11	112.03 (13)
O8—C8—H8B	109.3	C10—N4—C7	108.96 (12)
C7—C8—H8B	109.3	C11—N4—C7	108.03 (13)
H8A—C8—H8B	108.0	C12—N5—H5C	109.9 (13)
O8—C9—C10	111.11 (16)	C12—N5—H5A	108.4 (13)
O8—C9—H9A	109.4	H5C—N5—H5A	106.8 (17)
C10—C9—H9A	109.4	C12—N5—H5B	111.1 (13)
O8—C9—H9B	109.4	H5C—N5—H5B	111.2 (18)
C10—C9—H9B	109.4	H5A—N5—H5B	109.2 (18)
H9A—C9—H9B	108.0	C8—O8—C9	108.77 (14)
N4—C10—C9	111.04 (14)	H9D—O9—H9C	112 (2)
			
O1—C1—C2—C3	177.25 (14)	C1—C6—N1—O3	−136.51 (16)
C6—C1—C2—C3	0.8 (2)	C5—C6—N1—O2	−139.62 (17)
O1—C1—C2—N3	0.9 (2)	C1—C6—N1—O2	43.8 (2)
C6—C1—C2—N3	−175.60 (12)	C5—C4—N2—O4	1.0 (2)
C1—C2—C3—C4	0.8 (2)	C3—C4—N2—O4	179.69 (15)
N3—C2—C3—C4	177.14 (13)	C5—C4—N2—O5	−178.77 (14)
C2—C3—C4—C5	−2.5 (2)	C3—C4—N2—O5	−0.1 (2)
C2—C3—C4—N2	178.84 (12)	C3—C2—N3—O7	129.37 (17)
C3—C4—C5—C6	2.6 (2)	C1—C2—N3—O7	−54.0 (2)
N2—C4—C5—C6	−178.78 (13)	C3—C2—N3—O6	−51.9 (2)
C4—C5—C6—C1	−0.9 (2)	C1—C2—N3—O6	124.75 (16)
C4—C5—C6—N1	−177.21 (13)	C9—C10—N4—C11	−173.52 (13)
O1—C1—C6—C5	−177.07 (14)	C9—C10—N4—C7	−54.04 (18)
C2—C1—C6—C5	−0.7 (2)	C12—C11—N4—C10	−56.71 (17)
O1—C1—C6—N1	−0.8 (2)	C12—C11—N4—C7	−176.74 (13)
C2—C1—C6—N1	175.61 (13)	C8—C7—N4—C10	53.41 (19)
N4—C7—C8—O8	−58.1 (2)	C8—C7—N4—C11	175.35 (14)
O8—C9—C10—N4	58.90 (19)	C7—C8—O8—C9	60.6 (2)
N4—C11—C12—N5	−61.64 (18)	C10—C9—O8—C8	−60.8 (2)
C5—C6—N1—O3	40.0 (2)		

### Hydrogen-bond geometry (Å, º)

**Table:**

*D*—H···*A*	*D*—H	H···*A*	*D*···*A*	*D*—H···*A*
C7—H7*B*···O7^i^	0.97	2.55	3.465 (2)	158
C10—H10*B*···O6^ii^	0.97	2.63	3.503 (3)	150
C12—H12*A*···O2^iii^	0.97	2.55	3.349 (3)	139
N5—H5*C*···O9	0.89 (2)	1.86 (2)	2.741 (2)	172 (2)
N5—H5*A*···O1^iii^	0.89 (2)	1.89 (2)	2.777 (2)	172 (2)
N5—H5*B*···O5^iv^	0.85 (2)	2.35 (2)	3.054 (2)	142 (2)
N5—H5*B*···O9^v^	0.85 (2)	2.48 (2)	3.048 (2)	126 (2)
O9—H9*D*···N4^v^	0.84 (2)	1.98 (2)	2.7722 (19)	158 (2)
O9—H9*C*···O1	0.82 (2)	1.94 (2)	2.7196 (19)	160 (2)

Symmetry codes: (i) *x*−1, *y*, *z*; (ii) −*x*+1, −*y*+1, −*z*+1; (iii) −*x*+1, −*y*, −*z*+1; (iv) *x*−1, *y*−1, *z*−1; (v) −*x*, −*y*, −*z*+1.
